# Effects of Oral L-Carnitine on Liver Functions after Transarterial Chemoembolization in Intermediate-Stage HCC Patients

**DOI:** 10.1155/2015/608216

**Published:** 2015-11-19

**Authors:** Abeer Hassan, Yasuhiro Tsuda, Akira Asai, Keisuke Yokohama, Ken Nakamura, Tetsuya Sujishi, Hideko Ohama, Yusuke Tsuchimoto, Shinya Fukunishi, Usama M. Abdelaal, Usama A. Arafa, Ali T. Hassan, Ali M. Kassem, Kazuhide Higuchi

**Affiliations:** ^1^Second Department of Internal Medicine, Osaka Medical College, 2-7 Daigakumachi, Takatsuki, Osaka 569-8686, Japan; ^2^Department of Internal Medicine, Sohag Faculty of Medicine, Sohag University, Sohag 82524, Egypt

## Abstract

Transarterial chemoembolization (TACE) is usually followed by hepatic dysfunction. We evaluated the effects of L-carnitine on post-TACE impaired liver functions. *Methods*. 53 cirrhotic hepatocellular carcinoma patients at Osaka Medical College were enrolled in this study and assigned into either L-carnitine group receiving 600 mg oral L-carnitine daily or control group. Liver functions were evaluated at pre-TACE and 1, 4, and 12 weeks after TACE. *Results*. The L-carnitine group maintained Child-Pugh (CP) score at 1 week after TACE and exhibited significant improvement at 4 weeks after TACE (*P* < 0.01). Conversely, the control group reported a significant CP score deterioration at 1 week (*P* < 0.05) and 12 weeks after TACE (*P* < 0.05). L-carnitine suppressed serum albumin deterioration at 1 week after TACE. There were significant differences between L-carnitine and control groups regarding mean serum albumin changes from baseline to 1 week (*P* < 0.05) and 4 weeks after TACE (*P* < 0.05). L-carnitine caused prothrombin time improvement from baseline to 1, 4 (*P* < 0.05), and 12 weeks after TACE. Total bilirubin mean changes from baseline to 1 week after TACE exhibited significant differences between L-carnitine and control groups (*P* < 0.05). The hepatoprotective effects of L-carnitine were enhanced by branched chain amino acids combination. *Conclusion*. L-carnitine maintained and improved liver functions after TACE.

## 1. Introduction

Hepatocellular carcinoma (HCC) is the fifth most common cancer worldwide and the third most common cause of cancer-related deaths [1]. Furthermore, HCC is currently the leading cause of death among patients with liver cirrhosis [2].

Intermediate-stage HCC is defined as an extensive multifocal disease without vascular invasion in patients with preserved liver functions and absence of cancer-related symptoms. Transarterial chemoembolization (TACE) is considered the standard treatment for intermediate-stage HCC [3].

Hepatic failure after TACE is expected even in patients with relatively good hepatic reserve, significantly impairing the outcome of TACE, including patient survival [4, 5]. The maintenance of hepatic functional reserve is a major concern in patients with HCC who, in general, are treated repeatedly with TACE [6].

L-carnitine (4-*N*-trimethyl ammonium 3-hydroxybutyric acid) is a conditionally essential amino acid synthesized from essential amino acids methionine and lysine in human liver, kidneys, and brain but principally obtained from diet [7]. L-carnitine functions by transferring long-chain fatty acids across the mitochondrial membrane, enabling the oxidative release of energy [8]. L-carnitine deficiency is associated with liver cirrhosis because of limited dietary intake, absorption, and endogenous hepatic synthesis [9].

The protective role of L-carnitine against hepatotoxicity has been proposed in many studies [10–12]. Oral L-carnitine supplementation improved liver functions and histological patterns in patients with nonalcoholic steatohepatitis (NASH) [13]. Cirrhotic patients with minimal hepatic encephalopathy achieved improved quality of life with L-carnitine supplementation [14]. L-carnitine was an effective adjuvant to interferon and ribavirin in patients with chronic hepatitis C viral infection (HCV) [15].

Branched-chain amino acids (BCAA) are essential amino acids and include L-valine, L-leucine, and L-isoleucine. Several studies demonstrated that BCAA nutritional therapy decreased the risk of hepatic failure and improved general outcome in HCC patients undergoing variable treatment options [16].

In this study, we examined the protective effects of L-carnitine regarding liver dysfunction after TACE in intermediate-stage HCC patients when administered alone or in combination with BCAA.

## 2. Patients and Methods

### 2.1. Patients

The study included 53 HCC patients who underwent TACE between December 2012 and November 2013 at Osaka Medical College Hospital. All patients provided written informed consent to participate in the study. Study protocol conformed to the ethical guidelines of the Declaration of Helsinki (1975) and was approved by the Osaka Medical College ethical review committee. All patients were Japanese and had liver cirrhosis diagnosed by abdominal ultrasound and liver functions tests. HCC diagnosis was based on data obtained by contrast-enhanced computed tomography and hepatic-artery angiography. No patients had vessel invasion at the time of study enrollment, and none had been prescribed L-carnitine supplements before enrollment to the study.

### 2.2. Study Design

The study was prospective, and the patients were randomly assigned into two groups; the L-carnitine group included twenty-seven consecutive HCC patients who received a 300 mg tablet of L-carnitine twice daily starting from 2 weeks before TACE to week 12 after TACE. The control group included 26 consecutive HCC patients who did not receive L-carnitine supplementation. Thirty-one of the study patients had already been supplemented by late evening snacks of BCAA granules (LIVACT granules containing L-isoleucine 952 mg, L-leucine 1,904 mg, and L-valine 1,144 mg) prior to enrolment in this study and continued throughout study duration (we identified these patients as BCAA+ patients) while 22 patients did not receive BCAA granules (identified as BCAA−).

All patients were followed up in Osaka Medical College Hospital including clinical follow-up and laboratory measurements. The primary end point was improvement in Child-Pugh (CP) score and serum albumin. The secondary end point included improvement in other liver functions.

### 2.3. TACE Protocol

TACE for HCC was performed in conformity with Japanese guidelines by catheterization via femoral artery with superselective cannulation to the HCC hepatic feeding artery [17]. The infused agent was an emulsion of 50 mg of cisplatin (IA-call, Nihon-Kayaku) or 50 mg of farmorubicin (epirubicin hydrochloride, Pfizer) or other anticancer agents and 5 mL of Lipiodol (iodine addition products of ethyl esters of fatty acids obtained from poppy seed oil; Mitsui, Japan). The amount of emulsion was determined by the operator.

### 2.4. Laboratory Measurements

Laboratory measurements were performed 2 weeks before and 1, 4, and 12 weeks after TACE. Ascites was diagnosed by computed tomography or ultrasound. Laboratory measurements included serum albumin, total bilirubin, prothrombin time (PT), alanine aminotransferase (ALT), gamma-glutamyl transpeptidase (GGTP), and aspartate aminotransferase (AST) in addition to calculation of CP scores.

### 2.5. Statistical Analysis

Data were analyzed using SPSS version 22. Differences in laboratory data between study groups and differences within groups were analyzed using the Mann-Whitney *U* test and the *t*-test. Chi-square test was used for categorical data analysis. Probability (*P*) values <0.05 indicate statistical significance.

## 3. Results

### 3.1. Patients Characteristics

A total of 53 patients with HCC were enrolled in the current study. Three patients were withdrawn from the study because of non-liver-related deaths. There were no statistically significant differences between the two groups regarding demographic characteristics, tumor staging, and anticancer drugs used during TACE and baseline laboratory tests (Table 1). Similarly, no such differences were observed between L-carnitine and control groups in BCAA+ patients and BCAA− patients.

### 3.2. Effects of L-Carnitine on CP Score

**Figure 1:**
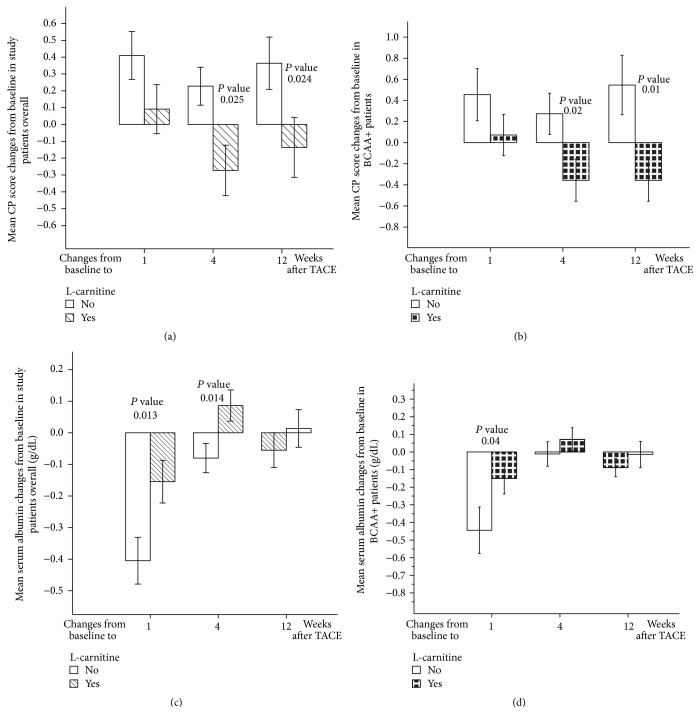
L-carnitine effects on CP score and serum albumin. (a) L-carnitine effects on mean CP score changes from baseline in study patients overall. (b) L-carnitine effects on mean CP score changes from baseline in BCAA+ patients. (c) L-carnitine effects on mean serum albumin changes from baseline in study patients overall. (d) L-carnitine effects on mean serum albumin changes from baseline in BCAA+ patients. CP: Child-Pugh; TACE: transarterial chemoembolization. Error bars represent standard errors.

In the L-carnitine group in this study, CP scores showed a nonsignificant deterioration at 1 week after TACE. CP scores showed significant improvement at 4 weeks after TACE (*P* < 0.01 compared with 1 week after TACE scores); CP scores at 4 and 12 weeks after TACE were better than baseline scores (Table 2). In contrast, the control group experienced a significant deterioration in CP scores at 1 week after TACE (*P* < 0.05 compared to baseline), reaching CP scores at 4 weeks after TACE worse than at baseline; CP scores at 12 weeks after TACE were significantly worse than baseline scores (*P* < 0.05) (Table 2). There were significant differences between the L-carnitine and control groups in CP score mean changes from baseline to 4 and 12 weeks after TACE (*P* < 0.05) (Figure 1(a)).

To investigate the combined effects of BCAA granules and L-carnitine, we evaluated BCAA+ and BCAA− patients. L-carnitine group in BCAA+ patients achieved significant CP score improvement from week 1 to week 4 after TACE (*P* < 0.05) (Table 3). There were significant differences between the L-carnitine and control groups in mean CP score changes from baseline to 4 weeks after TACE (*P* < 0.05) and 12 weeks after TACE (*P* < 0.05) (Figure 1(b)). L-carnitine group of BCAA− patients showed no significant changes in CP scores (Table 4). Comparison between the L-carnitine and the control groups revealed no significant differences in BCAA− patients.

### 3.3. Effects of L-Carnitine on Synthetic Liver Functions Tests

#### 3.3.1. Effects of L-Carnitine on Serum Albumin

In the L-carnitine group, serum albumin had significantly decreased by the first week after TACE (*P* < 0.05); serum albumin had significantly improved at 4 weeks after TACE (*P* < 0.05 compared to baseline and *P* < 0.01 compared to week 1 after TACE). Moreover, serum albumin at 12 weeks after TACE was higher than baseline and significantly higher than albumin level at 1 week after TACE (*P* < 0.05) (Table 2).

Nevertheless, in the control group, serum albumin had significantly decreased by the first week after TACE (*P* < 0.01); serum albumin was kept at levels lower than baseline at 4 weeks and 12 weeks after TACE (Table 2). Comparison between the L-carnitine and control groups regarding the means of serum albumin changes from baseline to 1 week and 4 weeks after TACE displayed significant differences (*P* < 0.05) (Figure 1(c)).

At 4 and 12 weeks following TACE, the L-carnitine group in BCAA+ patients had serum albumin levels higher than baseline values. Conversely, in the control group, serum albumin levels had significantly declined at 1 week after TACE (*P* < 0.01); serum albumin levels at 12 weeks after TACE were lower than at baseline (Table 3). There were significant differences between the L-carnitine and control groups in mean serum albumin decline from baseline to 1 week after TACE (*P* < 0.05) (Figure 1(d)). In BCAA− patients, serum albumin at 1 week after TACE showed a significant decline from baseline in the control group (*P* < 0.01) (Table 4). However, no significant differences were observed between the L-carnitine and control groups.

#### 3.3.2. Effects of L-Carnitine on PT

**Figure 2:**
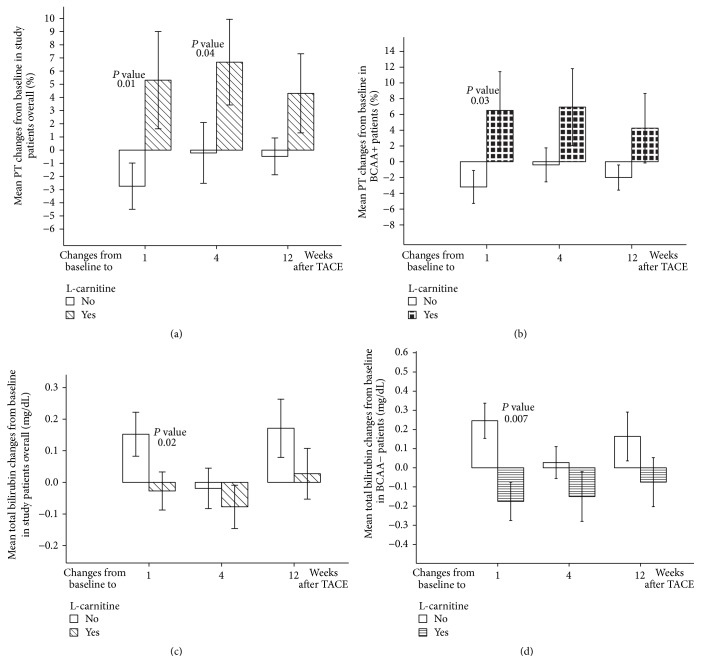
L-carnitine effects on PT and total bilirubin. (a) L-carnitine effects on mean PT changes from baseline in study patients overall. (b) L-carnitine effects on mean PT changes from baseline in BCAA+ patients. (c) L-carnitine effects on mean total bilirubin changes from baseline in study patients overall. (d) L-carnitine effects on mean total bilirubin changes from baseline in BCAA− patients. Footnote: PT: prothrombin time; TACE: transarterial chemoembolization. Error bars represent standard errors.

In the L-carnitine group, PT was elevated at week 1 after TACE and significantly higher than baseline at week 4 after TACE (*P* < 0.05). L-carnitine maintained better PT values at week 12 after TACE compared with baseline values (Table 2). Conversely, the control group displayed a significant PT decline at week 1 following TACE (*P* < 0.05) (Table 2). There were significant differences between the L-carnitine and control groups in mean PT changes from baseline to 1 week after TACE (*P* < 0.05) and 4 weeks after TACE (*P* < 0.05) (Figure 2(a)).

At 1, 4, and 12 weeks after TACE, L-carnitine group of BCAA+ patients reported higher PT values compared with baseline. Conversely, the control group demonstrated a significant PT decline at 1 week after TACE (*P* < 0.05); PT values at 12 weeks after TACE were lower than baseline values (Table 3). There were also significant differences between the L-carnitine and control groups in PT mean changes from baseline to 1 week after TACE (*P* < 0.05) (Figure 2(b)). In BCAA− patients, L-carnitine maintained PT values higher than at baseline at all follow-up intervals (Table 4), but there were no significant differences between the groups.

#### 3.3.3. Effects of L-Carnitine on Total Bilirubin

The current study demonstrated that L-carnitine prevented total bilirubin elevation at 1 week after TACE; total bilirubin at 12 weeks after TACE showed nonsignificant elevation over baseline values (Table 2). On the contrary, the control group exhibited total bilirubin levels significantly higher than baseline at the first week after TACE (*P* < 0.01). Furthermore, total bilirubin at week 12 after TACE was significantly higher than at baseline (*P* < 0.05) and at 4 weeks after TACE (*P* < 0.05) (Table 2). A comparison between the L-carnitine and control groups revealed significant differences in mean total bilirubin changes from baseline to 1 week after TACE (*P* < 0.05) (Figure 2(c)).

In BCAA+ patients, we observed no significant differences between L-carnitine and control groups (Table 3). In BCAA− patients, L-carnitine maintained total bilirubin at levels lower than baseline level at 1, 4, and 12 weeks after TACE; on the contrary, total bilirubin levels at week 1 after TACE were significantly higher than baseline level in the control group (*P* < 0.05) (Table 4). Comparison between the L-carnitine and control groups revealed significant differences in mean total bilirubin changes from baseline to 1 week after TACE (*P* < 0.01) (Figure 2(d)).

### 3.4. Effect of L-Carnitine on Liver Enzymes

A week after TACE, the control group demonstrated a significant rise in ALT (*P* < 0.05). However, L-carnitine limited ALT elevation at 1 week following TACE (Table 2). In the control group, GGTP levels were significantly higher than baseline levels at week 1 (*P* < 0.05) and week 4 after TACE (*P* < 0.01) (Table 2). Conversely, in the L-carnitine group, elevated GGTP levels did not differ significantly from baseline levels (Table 2). There were no significant changes in AST patterns in this study (Table 2). In the control group of BCAA+ and BCAA− patients, GGTP levels were significantly higher than baseline levels at week 4 after TACE (*P* < 0.01); L-carnitine did not affect ALT and AST patterns in both BCAA+ and BCAA− patients (Tables 3 and 4).

### 3.5. Effects of L-Carnitine on Cirrhotic Symptoms

The protective effects of L-carnitine in preventing occurrence and deterioration of ascites were shown at follow-up of study patients. In L-carnitine group, the number of ascites patients was fewer than baseline at 1 week after TACE and no ascites was detected at 4 weeks after TACE. On the contrary, the number of ascites patients in control group elevated at all follow-up periods with increased observation of massive ascites (Table 2).

Similar results were shown in L-carnitine and control groups in both BCAA+ patients and BCAA− patients (Tables 3 and 4).

In this study, none of study patients developed hepatic encephalopathy after TACE.

### 3.6. Subgroup Analysis of Effects of BCAA on Liver Functions after TACE

The previously mentioned follow-up results of CP score, serum albumin, and PT demonstrated the additive beneficial effects of combining BCAA and L-carnitine. Patients who received combination of L-carnitine and BCAA achieved better post-TACE liver functions compared to patients who received monotherapy of BCAA or L-carnitine (Tables 3 and 4).

Subgroup analysis of data obtained from BCAA+ and BCAA− patients in the current study irrespective of L-carnitine therapy revealed that BCAA+ patients achieved improvement of CP score at 4 weeks after TACE compared to baseline and significant improvement compared to 1 week after TACE (*P* < 0.05), while no significant improvement was observed in BCAA− patients (Table 5).

BCAA+ patients achieved significant improvement of serum albumin at 4 and 12 weeks after TACE (*P* < 0.001 and *P* < 0.05, resp., compared to 1 week after TACE). On the other hand, less significant improvement of serum albumin was shown in BCAA− patients at 4 and 12 weeks after TACE (*P* < 0.01and *P* < 0.05, resp., compared to 1 week after TACE) (Table 5).

Moreover, patients who did not receive L-carnitine or BCAA showed highly significant deterioration of serum albumin at 1 week after TACE compared to baseline (*P* < 0.001) and showed PT deterioration at 4 weeks after TACE compared to baseline and 1 week after TACE (Table 4). Conversely, patients who received BCAA alone achieved less significant deterioration of serum albumin at 1 week after TACE (*P* < 0.01) and accomplished PT improvement at 4 weeks after TACE compared to baseline and 1 week after TACE (Table 3).

Total bilirubin showed significant elevation at 1 week after TACE compared to baseline in patients who received neither BCAA nor L-carnitine therapy while no significant elevation was reported in patients who received BCAA (Tables 4 and 3).

## 4. Discussion

Hepatic failure after TACE is expected and is proportional to pre-TACE synthetic liver functions status [18]. In this study, L-carnitine exhibited hepatoprotective effects following TACE evinced by accomplishing improved CP scores and preventing deterioration of serum albumin, total bilirubin, and PT. To the best of our knowledge, this is the first study evaluating the effects of L-carnitine on liver functions following TACE. The current study clarified early and late post-TACE improvement in liver functions by L-carnitine therapy.

The present study reported very beneficial effects of L-carnitine on CP score showing a significant improvement at 4 and 12 weeks following TACE. These effects may be attributed to the combination of the favorable L-carnitine effects on serum albumin, PT, total bilirubin, and ascites. As regards ascites, better results were observed in L-carnitine group compared to control group that may be justified by less deteriorated serum albumin profile after TACE with L-carnitine intake.

L-carnitine plays an important role in energy production by transporting long-chain fatty acids across mitochondrial membranes in skeletal muscles [19]. Cirrhotic patients are invariably deficient in L-carnitine [9]. This L-carnitine deficiency inhibits fatty acid mobilization and hence oxidation for energy in skeletal muscles; skeletal muscles are presumed to utilize BCAA as a substitute for fatty acids to produce energy. Cirrhotic patients already suffer from shortage in BCAA that are required for albumin synthesis [20]. BCAA usage as energy source in skeletal muscles adds an additional burden to cirrhotic patients, rendering them more albumin deficient. L-carnitine supplementations correct L-carnitine deficiency; thus, it may preserve BCAA for albumin synthesis and improve serum albumin profiles. In the current study, L-carnitine suppressed early deterioration of serum albumin and maintained its levels better than at baseline.

In a similar fashion, Malaguarnera and his colleagues, in a study evaluating L-carnitine as an adjuvant therapy for interferon plus ribavirin-treated HCV patients, demonstrated that L-carnitine inhibited serum albumin decline at the end of treatment course [15]. Conversely, there was no significant effect of L-carnitine supplementation for 6 months on serum albumin levels in NASH patients [13]. Mean baseline serum albumin levels in L-carnitine-supplemented NASH patients (4.7 ± 0.5 g/dL) were optimal and much better than mean baseline levels in L-carnitine-supplemented cirrhotic patients in our study (3.2 ± 0.5 g/dL); that difference may explain why L-carnitine had no effects on serum albumin levels in patients with NASH.

L-carnitine in this study had improved PT at all follow-up intervals. These finding are in concordance with the effects of L-carnitine on PT reported in children with acute lymphoblastic leukemia receiving chemotherapy [12]. In addition, L-carnitine improved total bilirubin profiles, resembling the observed effect of L-carnitine on total bilirubin in hepatic encephalopathy patients [14]. Conversely, L-carnitine showed no effects on total bilirubin in interferon plus ribavirin-treated patients with HCV [15]. This controversy may be due to long duration of follow-up for HCV patients as total bilirubin was evaluated at 12 months after treatment initiation, while the adverse effect of ribavirin elevating total bilirubin because of hemolysis was usually observed within 3 months from treatment initiation [21].

The favorable effects of L-carnitine on PT and total bilirubin may be due to its ability to relieve hepatic oxidative stress [22]. L-carnitine significantly improved liver antioxidant capacity in cisplatin-treated rats by increasing the Glutathione pool, blockage of the free radical production [23], and regulation of peroxisome proliferator-activated receptor alpha [22].

HCC patients undergoing variable treatment options earned many benefits from BCAA oral therapy [16]. BCAA granules therapy for decompensated cirrhotic patients revealed improvement in event-free survival, quality of life, and serum albumin concentrations [20]. BCAA oral intake improved functions of hepatic parenchymal cells in cirrhotic patients [24]. Moreover, Nishikawa et al. reported that BCAA granules significantly suppressed the deterioration in hepatic functional reserve (serum albumin and CP score) at 3 and 6 months after TACE [25].

In our study, L-carnitine combination with BCAA revealed more pronounced effects on CP scores, serum albumin, and PT than observed effects in BCAA− patients. We found no available data of previous studies evaluating BCAA and L-carnitine combination therapy. This study is also the first report of the combination effect of L-carnitine and BCAA on liver functions.

On the other hand, comparison between BCAA+ and BCAA− patients regardless of L-carnitine therapy revealed lesser post-TACE deterioration of CP score, serum albumin, and liver enzymes in BCAA+ patients.

The beneficial effects of BCAA therapy without L-carnitine intake were more cleared up in the present study by analyzing data obtained from patients who received neither BCAA nor L-carnitine compared to patients who received BCAA alone. These data demonstrated favorable effects of BCAA on serum albumin, PT, and total bilirubin.

There were no adverse effects by L-carnitine intake in any of study patients.

This study has the following limitations: (1) The relatively small number of patients may inflate the beneficial effects of L-carnitine on liver functions after TACE. (2) Oral administration may decrease the L-carnitine efficacy as it has low oral bioavailability and poor absorption in cirrhotic patients. Parenteral administration is more effective but decreases patient tolerability.

In conclusion, L-carnitine maintained and improved liver functions following TACE. The hepatoprotective effects of L-carnitine in this study were enhanced by BCAA granules combination. L-carnitine and BCAA combination therapy may be offered as a new liver support tool in patients with HCC.

## Figures and Tables

**Table 1:** Baseline clinical and laboratory data.

Parameter (mean ± SD)	L-carnitine group (*N* = 24)	Control group (*N* = 26)	*P*
Sex (male/female)	17/7	21/5	0.5
Age	71.6 ± 7.6	72.3 ± 6.8	0.7
CP score	6.04 ± 1.04	5.88 ± 0.99	0.4
Stage (2/3/4)	7/15/2	9/15/2	0.9
Drug (cisplatin/others)	18/6	21/5	0.3
BCAA (yes/no)	16/8	12/14	0.1
S. albumin (g/dL)	3.22 ± 0.57	3.35 ± 0.56	0.2
PT (%)	86.7 ± 19.4	89.2 ± 17	0.5
T. bilirubin (mg/dL)	1.02 ± 0.57	0.93 ± 0.46	0.4
ALT (U/L)	32.6 ± 17.6	41.8 ± 28.8	0.3
AST (U/L)	51.5 ± 25.9	57 ± 37.8	0.7
GGTP (U/L)	70.7 ± 80.9	66.3 ± 67.3	0.8
Ascites (no/moderate/massive)	21/3/0	21/5/0	0.2
Encephalopathy (yes/no)	24/0	26/0	0.6

SD: standard deviation; CP: Child-Pugh; BCAA: branched-chain amino acids; S. albumin: serum albumin; PT: prothrombin time; T. bilirubin: total bilirubin; ALT: alanine aminotransferase; AST: aspartate aminotransferase; GGTP: gamma-glutamyl transpeptidase.

**Table 2:** Effects of L-carnitine in overall study patients.

	Parameter (mean ± SD)	Pretreatment	After TACE		
			1 week	4 weeks	12 weeks
Control group (*N* = 26)	CP score	5.88 ± 0.99	6.24 ± 1.05^†*∗*^	6.04 ± 1.07	6.39 ± 1.55^†*∗*^
	S. albumin (g/dL)	3.35 ± 0.56	2.94 ± 0.57^†*∗∗∗*^	3.29 ± 0.58^‡*∗∗∗*^	3.21 ± 0.63^‡*∗∗*^
	PT (%)	89.2 ± 17	85.9 ± 16.9^†*∗*^	88.2 ± 17.7	87.1 ± 18.2
	T. bilirubin (mg/dL)	0.93 ± 0.46	1.13 ± 0.51^†*∗*^	0.96 ± 0.55^‡*∗*^	1.21 ± 0.7^†*∗*,§*∗*^
	ALT (IU/L)	41.8 ± 28.8	56.2 ± 31^†*∗*^	38.7 ± 21.1^‡*∗*^	37.6 ± 18.8
	AST (IU/L)	57 ± 37.8	50.8 ± 26.5	55.3 ± 31.6	60.3 ± 44.3
	GGTP (IU/L)	66.3 ± 67.3	84.5 ± 100.3^†*∗*^	91.8 ± 84.7^†*∗∗∗*^	59.9 ± 74.7
	Ascitis (no/moderate/massive)	21/5/0	20/5/1	19/5/2	17/5/4
	Encephalopathy (no/yes)	26/0	26/0	26/0	26/0

L-carnitine group (*N* = 24)	CP score	6.04 ± 1.04	6.17 ± 0.86	5.75 ± 0.73^‡*∗∗*^	5.91 ± 0.92
	S. albumin (g/dL)	3.22 ± 0.57	3.05 ± 0.46^†*∗*^	3.33 ± 0.5^†*∗*,‡*∗∗*^	3.26 ± 0.49^‡*∗*^
	PT (%)	86.7 ± 19.4	91.5 ± 18.4	93.3 ± 16.8^†*∗*^	90.7 ± 16.4
	T. bilirubin (mg/dL)	1.02 ± 0.57	0.97 ± 0.55	0.93 ± 0.46	1.05 ± 0.63
	ALT (IU/L)	32.6 ± 17.6	41.7 ± 23.1^†*∗*^	34.7 ± 20.8	33.9 ± 11.6
	AST (IU/L)	51.5 ± 25.9	50.2 ± 32.1	54.5 ± 28	49.4 ± 15.6
	GGTP (IU/L)	70.7 ± 80.9	91.4 ± 124.4	97.1 ± 148.6	61.8 ± 43.4
	Ascitis (no/moderate/massive)	21/3/0	22/2/0	24/0/0	23/0/1
	Encephalopathy (no/yes)	24/0	24/0	24/0	24/0

SD: standard deviation; CP: Child-Pugh; S. albumin: serum albumin; PT: prothrombin time; T. bilirubin: total bilirubin; ALT: alanine aminotransferase; AST: aspartate aminotransferase; GGTP: gamma-glutamyl transpeptidase.

^†^Significant difference compared with baseline; ^‡^significant difference compared with 1 week after TACE; ^§^significant difference compared with 4 weeks after TACE; ^*∗*^ *P* < 0.05; ^*∗∗*^ *P* < 0.01; ^*∗∗∗*^ *P* < 0.001.

**Table 3:** Effects of L-carnitine in BCAA+ patients.

	Parameter (mean ± SD)	Pretreatment	After TACE		
			1 week	4 weeks	12 weeks
Control group (*N* = 12)	CP score	6 ± 1.04	6.5 ± 1.16	6.25 ± 1.28	6.64 ± 1.69
	S. albumin (g/dL)	3.25 ± 0.52	2.81 ± 0.55^†*∗∗*^	3.25 ± 0.51^‡*∗∗*^	3.01 ± 0.52
	PT (%)	87.4 ± 17.3	83.4 ± 19.1^†*∗*^	88.4 ± 18.3	83.6 ± 19.8
	T. bilirubin (mg/dL)	1.1 ± 0.58	1.2 ± 0.55	1.05 ± 0.63	1.41 ± 0.63
	ALT (IU/L)	43.5 ± 19.3	56.5 ± 26.6	42.9 ± 19.8	38.4 ± 11.4
	AST (IU/L)	52.4 ± 20.4	45.8 ± 9	50 ± 21	52.7 ± 21.1
	GGTP (IU/L)	80.4 ± 93.9	104.3 ± 140.5	116.3 ± 120^†*∗∗*^	43.9 ± 32.3
	Ascitis (no/moderate/massive)	8/4/0	8/3/1	6/4/2	6/4/2
	Encephalopathy (no/yes)	12/0	12/0	12/0	12/0

L-carnitine group (*N* = 16)	CP score	6.25 ± 1.6	6.38 ± 0.88	5.88 ± 0.8^‡*∗*^	5.93 ± 0.99
	S. albumin (g/dL)	3.13 ± 0.61	2.97 ± 0.42	3.25 ± 0.56^‡*∗∗*^	3.16 ± 0.49
	PT (%)	87.2 ± 20.7	92.3 ± 16.1	94.1 ± 18.2	91.6 ± 14.2
	T. Bilirubin (mg/dL)	1.01 ± 0.62	1.02 ± 0.62	0.95 ± 0.53	1.1 ± 0.78
	ALT (IU/L)	32.2 ± 12.5	42.7 ± 22.6	30.6 ± 8.3	36.1 ± 11
	AST (IU/L)	51.7 ± 25.3	51.2 ± 31.9	51.3 ± 18.7	53.5 ± 16.8
	GGTP (IU/L)	62 ± 53.7	72.8 ± 56.6	70.6 ± 36.8	60.4 ± 38.5
	Ascitis (no/moderate/massive)	14/2/0	14/2/0	16/0/0	15/0/1
	Encephalopathy (no/yes)	16/0	16/0	16/0	16/0

SD: standard deviation; CP: Child-Pugh; S. albumin: serum albumin; PT: prothrombin time; T. bilirubin: total bilirubin; ALT: alanine aminotransferase; AST: aspartate aminotransferase; GGTP: gamma-glutamyl transpeptidase; TACE: transarterial chemoembolization.

^†^Significant difference compared with baseline; ^‡^significant difference compared with 1 week after TACE; ^*∗*^ *P* < 0.05; ^*∗∗*^ *P* < 0.01.

**Table 4:** Effects of L-carnitine in BCAA− patients.

	Parameter (mean ± SD)	Pretreatment	After TACE		
			1 week	4 weeks	12 weeks
Control group (*N* = 14)	CP score	5.79 ± 0.97	6 ± 0.91	5.86 ± 0.86	6.17 ± 1.46
	S. albumin (g/dL)	3.42 ± 0.6	3.06 ± 0.59^†*∗∗∗*^	3.32 ± 0.66^‡*∗*^	3.37 ± 0.6^‡*∗∗*^
	PT (%)	90.8 ± 17.3	88.3 ± 15.1	88 ± 17.9	90.4 ± 16.9
	T. bilirubin (mg/dL)	0.78 ± 0.29	1.06 ± 0.49^†*∗*^	0.89 ± 0.48	1.05 ± 0.74
	ALT (IU/L)	40.3 ± 35	55.9 ± 35	35.1 ± 22	36.9 ± 24
	AST (IU/L)	60.9 ± 48	55.3 ± 35	59.7 ± 38	67.3 ± 58
	GGTP (IU/L)	54.2 ± 30	66.3 ± 35	72.6 ± 35^†*∗∗*^	74.5 ± 98
	Ascitis (no/moderate/massive)	13/1/0	12/2/0	13/1/0	11/1/2
	Encephalopathy (no/yes)	14/0	14/0	14/0	14/0

L-carnitine group (*N* = 8)	CP score	5.63 ± 0.91	5.75 ± 0.7	5.5 ± 0.53	5.88 ± 0.83
	S. albumin (g/dL)	3.38 ± 0.47	3.22 ± 0.52	3.5 ± 0.54	3.45 ± 0.48
	PT (%)	85.6 ± 17.7	89.7 ± 24.3	91.8 ± 14.8	89.2 ± 20.6
	T. bilirubin (mg/dL)	1.05 ± 0.48	0.87 ± 0.42	0.9 ± 0.34	0.97 ± 0.24
	ALT (IU/L)	33.5 ± 26.1	39.8 ± 25.7	43 ± 34.1	30 ± 12.3
	AST (IU/L)	51.1 ± 28.7	48.1 ± 34.6	61.1 ± 41.9	42.1 ± 10.4
	GGPT (IU/L)	88.1 ± 121	128.6 ± 204	150.1 ± 254	64.3 ± 53
	Ascitis (no/moderate/massive)	7/1/0	8/0/0	8/0/0	8/0/0
	Encephalopathy (no/yes)	8/0	8/0	8/0	8/0

SD: standard deviation; CP: Child-Pugh; S. albumin: serum albumin; PT: prothrombin time; T. bilirubin: total bilirubin; ALT: alanine aminotransferase; AST: aspartate aminotransferase; GGTP: gamma-glutamyl transpeptidase; TACE: transarterial chemoembolization.

^†^Significant difference compared with baseline value; ^‡^significant difference compared with 1 week after TACE; ^*∗*^ *P* < 0.05; ^*∗∗*^ *P* < 0.01; ^*∗∗∗*^ *P* < 0.001.

**Table 5:** Subgroup analysis in BCAA+ and BCAA− patients.

	Parameter (mean ± SD)	Pretreatment	After TACE		
			1 week	4 weeks	12 weeks
BCAA+ (*N* = 28)	CP score	6.14 ± 1.04	6.43 ± 0.99	6.04 ± 1.03^‡*∗*^	6.24 ± 1.36
	S. albumin (g/dL)	3.18 ± 0.56	2.90 ± 0.48^†*∗∗∗*^	3.25 ± 0.53^‡*∗∗∗*^	3.10 ± 0.49^‡*∗*^
	PT (%)	87.32 ± 18.99	88.50 ± 17.69	91.59 ± 18.19	88.13 ± 16.96
	T. bilirubin (mg/dL)	1.05 ± 0.59	1.10 ± 0.58	1.00 ± 0.56	1.22 ± 0.72
	ALT (IU/L)	37.07 ± 16.50	48.68 ± 24.95^†*∗*^	35.89 ± 15.43^‡*∗∗*^	37.16 ± 11.05^‡*∗*^
	AST (IU/L)	52.04 ± 22.97	48.93 ± 24.65	50.79 ± 19.39	53.20 ± 18.43
	GGTP (IU/L)	69.89 ± 72.74	86.36 ± 100.41^†*∗*^	89.26 ± 82.77	53.16 ± 36.19^§*∗*^

BCAA− group (*N* = 22)	CP score	5.73 ± 0.93	5.90 ± 0.83	5.73 ± 0.76	6.05 ± 1.23
	S. albumin (g/dL)	3.41 ± 0.55	3.12 ± 0.56^†*∗∗∗*^	3.38 ± 0.61^‡*∗∗*^	3.40 ± 0.60^‡*∗*^
	PT (%)	88.9 ± 17.2	88.8 ± 18.2	89.5 ± 16.5	89.9 ± 18.0
	T. bilirubin (mg/dL)	0.88 ± 0.38	0.99 ± 0.46	0.89 ± 0.43	1.02 ± 0.58
	ALT (IU/L)	37.86 ± 32.04	49.81 ± 32.60	38.00 ± 26.65	34.15 ± 20.24
	AST (IU/L)	57.36 ± 42.03	52.62 ± 34.73	60.27 ± 38.91	57.25 ± 46.66
	GGTP (IU/L)	66.59 ± 76.06	90.05 ± 127.71	100.82 ± 154.50	70.50 ± 82.04

SD: standard deviation; CP: Child-Pugh; S. albumin: serum albumin; PT: prothrombin time; T. bilirubin: total bilirubin; ALT: alanine aminotransferase; AST: aspartate aminotransferase; GGTP: gamma-glutamyl transpeptidase; TACE: transarterial chemoembolization.

^†^Significant difference compared with baseline value; ^‡^significant difference compared with 1 week after TACE; ^§^significant difference compared with 4 weeks after TACE; ^*∗*^ *P* < 0.05; ^*∗∗*^ *P* < 0.01; ^*∗∗∗*^ *P* < 0.001.
